# Olfactory bulb surroundings can help to distinguish Parkinson’s disease from non-parkinsonian olfactory dysfunction

**DOI:** 10.1016/j.nicl.2020.102457

**Published:** 2020-10-02

**Authors:** Cécilia Tremblay, Jie Mei, Johannes Frasnelli

**Affiliations:** a Department of Anatomy, Université du Québec à Trois-Rivières, 3351 Boul. des Forges, Trois-Rivières, Québec G9A 5H7, Canada; b Research Center, Sacré-Coeur Hospital of Montreal, 5400 boul. Gouin Ouest, Montréal, Québec H4J 1C5, Canada

**Keywords:** Parkinson’s disease, Olfactory dysfunction, Olfactory bulb volume, Machine learning, Convolutional neural networks

## Abstract

**Background:**

The olfactory bulb is one of the first regions of insult in Parkinson’s disease (PD), consistent with the early onset of olfactory dysfunction. Investigations of the olfactory bulb may, therefore, help early pre-motor diagnosis. We aimed to investigate olfactory bulb and its surrounding regions in PD-related olfactory dysfunction when specifically compared to other forms of non-parkinsonian olfactory dysfunction (NPOD) and healthy controls.

**Methods:**

We carried out MRI-based olfactory bulb volume measurements from T2-weighted imaging in scans from 15 patients diagnosed with PD, 15 patients with either post-viral or sinonasal NPOD and 15 control participants. Further, we applied a deep learning model (convolutional neural network; CNN) to scans of the olfactory bulb and its surrounding area to classify PD-related scans from NPOD-related scans.

**Results:**

Compared to controls, both PD and NPOD patients had smaller olfactory bulbs, when measured manually (both p < .001) whereas no difference was found between PD and NPOD patients. In contrast, when a CNN was used to differentiate between PD patients and NPOD patients, an accuracy of 88.3% was achieved. The cortical area above the olfactory bulb which stretches around and into the olfactory sulcus appears to be a region of interest in the differentiation between PD and NPOD patients.

**Conclusion:**

Measures from and around the olfactory bulb in combination with the use of a deep learning model may help differentiate PD patients from patients with NPOD, which may be used to develop early diagnostic tools based on olfactory dysfunction.

## Highlights

• Olfactory bulb inquiry might help to develop early markers of Parkinson’s disease (PD).
• Olfactory bulb volume is equally reduced in PD than in other olfactory dysfunctions.
• Machine learning yield an accuracy of 88% to distinguish PD-related olfactory loss.
• Olfactory bulb scans can help to distinguish PD-related olfactory dysfunction.

## 1 Introduction

Olfactory dysfunction (OD) is a highly prevalent symptom of Parkinson’s disease (PD) affecting over than 90% of PD patients (Doty, 2012, Haehner et al., 2009). OD appears well before the onset of motor symptoms and is therefore considered as an early marker of PD (Bowman, 2017, Ross et al., 2008). Even though the cause of OD in PD is still unknown, olfactory deficits have been associated with alterations of central olfactory relevant regions including the olfactory bulb, the first relay station of the olfactory system which is located under the orbitofrontal cortex (Braak et al., 2003a, Hawkes et al., 2010). Consistent with the early onset of OD, accumulation of Lewy bodies, a pathological hallmark of PD, starts in the olfactory bulb (Beach et al., 2009a, Braak et al., 2003a, Hawkes et al., 2007). Accordingly, post-mortem studies revealed a significant loss of neurons in the olfactory bulb of PD patients (Pearce et al., 1995). The olfactory bulb is considered as a possible induction site for Lewy pathology (Beach et al., 2009a, Beach et al., 2009b), and it may serve as an entry point for pathogens to enter and spread throughout the brain via the olfactory pathways (Braak et al., 2003b, Doty, 2008, Rey et al., 2018). Furthermore, pathological changes are observed across different brain regions of the olfactory system, including the anterior olfactory nucleus, the amygdala, the piriform, the entorhinal, and the orbitofrontal cortex (Harding et al., 2002, Silveira-Moriyama et al., 2009). Therefore, investigating olfactory structures, especially the olfactory bulb, may help early pre-motor diagnosis that might eventually contribute to halting or delaying disease progression (Berardelli et al., 2013, Fullard et al., 2017)*.*

Magnetic resonance imaging (MRI)-based measurements of the olfactory bulb are an established method commonly used to assess olfactory bulb volume as an indicator of olfactory function (Burmeister et al., 2011, Rombaux et al., 2009a, Yousem et al., 1997). Volumetric measures of the olfactory bulb as well as the olfactory sulcus, a cortical structure of the orbitofrontal cortex just above the olfactory bulb, are positively correlated with psychophysical measurements of olfactory function in health (Buschhuter et al., 2008, Hummel et al., 2003, Mazal et al., 2016) and disease (Haehner et al., 2008, Hummel et al., 2015). Olfactory bulb volume decreases with aging (Buschhuter et al., 2008, Yousem et al., 1998) and in patients with OD (Hummel et al., 2015), but increases during recovery from OD (Gudziol et al., 2009, Rombaux et al., 2012) or following olfactory training (Negoias et al., 2017), on account of its high plasticity (Huart et al., 2019, Lötsch et al., 2014). The olfactory bulb receives bottom-up projections from the nasal mucosa as the olfactory receptor neuron directly project to the olfactory bulb, and forwards information to various parts of the primary olfactory cortex including the anterior olfactory nucleus, the olfactory tubercle, the piriform cortex, the amygdala and the entorhinal cortex (Smith and Bhatnagar, 2019), that in turn project principally to the orbitofrontal cortex and other structures of the secondary olfactory cortex (Lundstrom et al., 2011). Here, the area around the olfactory sulcus equally exhibits correlations between anatomical measures (cortical thickness, depth) and olfactory function (Delon-Martin et al., 2013, Frasnelli et al., 2010, Hummel et al., 2003). In addition, the olfactory bulb can also be affected by top-down modulation due to projections from higher olfactory and non-olfactory cortical structures as different neuromodulatory fibers enter the olfactory bulb to inhibit or facilitate its activity and plays a role in olfactory learning and habituation (Cleland and Linster, 2019, Linster and Cleland, 2002, Rothermel and Wachowiak, 2014).

Measurements of the olfactory bulb volume of PD patients have led to mixed results. Some studies reported reduced olfactory bulb volume in PD as opposed to controls (Brodoehl et al., 2012, Chen et al., 2014, Hang et al., 2015, Tanik et al., 2016, Wang et al., 2011), whereas other found no significant group differences (Altinayar et al., 2014, Hakyemez et al., 2013, Hummel et al., 2010, Mueller et al., 2005a, Paschen et al., 2015). This may be related to low sample size, selected populations, and other factors, as a meta-analysis comprising 216 PD patients and 175 controls revealed a reduced olfactory bulb volume in PD patients compared with healthy controls, though putting forward the need for further investigations (Li et al., 2016). In analogy, the olfactory sulcus is also reduced in PD patients (Tanik et al., 2016, Wang et al., 2011), although there are important inconstancies across studies and others did not report any differences (Hang et al., 2015). Pathophysiology of PD is still poorly understood and several hypotheses might explain the reduced olfactory bulb volume in PD, including (a) mechanisms affecting bottom-up transfer of information by potential alterations of the olfactory epithelium, and (b) top-down mechanisms through alterations of the central nervous system or (c) direct affection of the olfactory bulb (Mazal et al., 2016).

OD is not specific to PD as there are several conditions that can affect olfactory function (Landis et al., 2004), and only a small proportion of patients with idiopathic OD will convert to PD (Haehner et al., 2019). Therefore, a step towards using OD as an early diagnostic tool in PD patients is to differentiate PD-related OD from other forms of acquired non-parkinsonian OD (NPOD), such as post-viral OD (i.e., following a viral infection of the upper respiratory tract), sinonasal OD (i.e., in the context of sinonasal disease), or post-traumatic OD (i.e., as a consequence of a traumatic brain injury). Olfactory bulb volume and olfactory sulcus depth have been extensively studied in different conditions affecting olfaction (Hummel et al., 2015) and a reduced olfactory bulb volume was reported in patients with post-viral OD (Mueller et al., 2005b, Rombaux et al., 2006a, Rombaux et al., 2009b, Yao et al., 2018), post-traumatic OD (Han et al., 2018, Mueller et al., 2005b, Rombaux et al., 2006b, Yousem et al., 1999), sinonasal OD (Rombaux et al., 2008, Shehata et al., 2018), idiopathic OD (Rombaux et al., 2010) and in OD related to nasal obstruction (Altundag et al., 2014, Askar et al., 2015). However, no studies have specifically compared the olfactory bulb structure of PD patients to that of patients with NPOD. While NPOD affects the olfactory bulb mainly by alterations of the olfactory epithelium or by direct damage to the olfactory bulb, PD may also influence the olfactory bulb via central alterations in a disease specific manner; we therefore hypothesized that the olfactory bulb exhibits different structural features in PD patients and patients with NPOD.

Although regarded as the current standard, manual measurement of the olfactory bulb from MR images is a technique that might present with some limitations when aiming at differentiating PD and NPOD patients as these measures (1) only take the olfactory bulb volume into account and do not consider its shape, or the surrounding area that might contain relevant information, (2) are largely dependent on experience and therefore are subject to variability and potential errors (Burmeister et al., 2011). Lately, alternative approaches such as deep learning models that can automatically and objectively extract information and patterns from structural and functional neuroimaging data, have been introduced. Advancements in deep learning led to diverse applications to medical image segmentation and classification for diagnostic purposes (Razzak et al., 2018). Some of these algorithms yield high performance in the diagnosis of early PD, and are considered as a promising tool for the search of biomarkers for neurologic diseases (Hirschauer et al., 2015, Karapinar, 2020, Parisi et al., 2018, Shinde et al., 2019). More specifically, convolutional neural networks (CNN), a class of artificial neural networks frequently used in image processing and classification (Krizhevsky et al., 2012), perform very well in the extraction of higher level features from medical images (Yasaka et al., 2018) and give rise to high accuracy in the detection and assessment of neurological conditions such as PD (Sivaranjini and Sujatha, 2019) or Alzheimer’s disease (Wang et al., 2018). Taken together, we hypothesize that the use of deep learning algorithms differentiates PD-related OD from NPOD based on structural characteristics of the olfactory bulb and/or its surrounding regions.

We therefore designed and conducted this study to specifically investigate olfactory bulb structural differences between PD patients in comparison to patients with other forms of NPOD. More specifically, we first aimed to assess MRI-based olfactory bulb volume manual measurements in PD patients when compared to NPOD patients and healthy matched controls. Secondly, we applied a deep learning model to MRI images of the olfactory bulb and its surrounding regions to assess its ability to differentiate PD-related OD from NPOD.

## 2 Methods

### 2.1 Participants

All aspects of the study were performed in accordance with the Declaration of Helsinki on biomedical research involving human subjects. The study protocol was approved by the local ethics committees (Research Center of the Institut Universitaire de Gériatrie de Montréal at University of Montreal and at the University of Quebec at Trois-Rivières). After being thoroughly informed about the study protocol, participants provided written consent prior to their inclusion in the study.

From the 48 participants initially enrolled, 45 participants completed the study, 2 participants were claustrophobic, one had a severe tremor and could not complete the scanning session. We included 15 PD patients that were recruited through the Quebec Parkinson Network, and were diagnosed with PD according to the UK PD Society Brain Bank diagnostic criteria (Litvan et al., 2012). Diagnosis ascertainment and clinical data were provided by the Quebec Parkinson Network (Gan-Or et al., 2020). All patients were on stable anti-parkinsonian medication. Hoehn and Yahr stage, age at onset, disease duration and calculated Levodopa equivalent daily doses (Tomlinson et al., 2010) are presented in Table 1. Participants with unclear diagnosis and/or symptoms of atypical parkinsonian syndrome were excluded, as were participants with nasal pathology that might have caused concurrent OD non-related to the disease (Hummel et al., 2017).

**Table 1:** Participants’ characteristics.

Variable	Controls (n = 15)	PD patients (n = 15)	NPOD patients (n = 15)	P values from between group analysis
Sex (F/M)	7;8	7;8	6;9	NA
Age	66.3 ± 6.3	66.8 ± 7.3	62.8 ± 9.2	p = .31
Female	65.8 ± 7.3	64.5 ± 6.5	60.7 ± 8.7	NA
Male	66.8 ± 5.6	68.9 ± 7.8	66.0 ± 3.0	NA
Age at onset (yrs)	NA	60.5 ± 7.8	NA	NA
Disease duration (yrs)	NA	6.3 ± 2.8	NA	NA
H&Y disease stage	NA	1.6 ± 0.6 (1–3)	NA	NA
LEDD (mg)	NA	527.0 ± 211.5	NA	NA
MoCA	27.4 ± 2.5	27.0 ± 2.8	27.2 ± 2.3	p = .96
BDI	1.2 ± 1.6	6.1 ± 3.4	2.3 ± 2.9	p < .001
TDI score	38.0 ± 3.0	17.5 ± 6.9	17.3 ± 7.7	p < .001
Threshold	10.4 ± 2.4	2.2 ± 1.5	3.0 ± 3.1	p < .001
Discrimination	12.6 ± 1.6	8.5 ± 2.7	7.2 ± 3.4	p < .001
Identification	13.5 ± 1.1	7.5 ± 3.5	7.5 ± 2.4	p < .001

Data are presented as means and standard deviation of the mean. Abbreviations: H&Y = Hoehn & Yahr Stage, LEDD = Levodopa equivalent daily dose, MoCA = Montreal Cognitive Assessment, BDI = Beck Depression Inventory, TDI = summation of scores of the 3 olfactory subtests (threshold, discrimination and identification).

Furthermore, we included 15 patients with NPOD that were recruited through the lab’s database. The probable cause of OD was subjectively evaluated in an interview with the patient using a questionnaire adapted from Hummel et al. (2017). Included patients had either post-viral (n = 10) or sinonasal OD (n = 5). We specifically excluded (a) participants with neurological conditions or signs of motor dysfunction, (b) patients with idiopathic OD as they may have an elevated risk of developing PD (Haehner et al., 2019), (c) post-traumatic OD as the condition may be associated with neural damages unrelated to the OD (Lotsch et al., 2016), and (d) congenital anosmia as the condition is associated with altered neuronal processing (Frasnelli et al., 2013).

Finally, we enrolled 15 control participants, in good general health with a normal olfactory function, from the community. Participants with neurological conditions or signs of motor dysfunction, cognitive decline or olfactory pathology were specifically excluded. Control participants were matched in terms of age and sex with PD patients. We confirmed that there was no age differences across the three groups [one-way ANOVA: F(2, 42) = 1.18; p = .31]. Further, cognitive function was assessed using the Montreal Cognitive Assessment (MoCA) (Nasreddine et al., 2005) and symptoms of depression were assessed using the Beck Depression Inventory (BDI) questionnaire (Beck et al., 1961) in all participants. Report to Table 1 for participant’s clinical data.

### 2.2 Olfactory testing

All participants underwent olfactory testing using standardized “Sniffin Sticks” test (Burghart, Wedel, Germany), including olfactory threshold, discrimination and identification tasks (Hummel et al., 1997, Hummel et al., 2007), for which we presented odorants in pen-like dispensing devices. Specifically, we assessed odor threshold with rose odor (phenyl ethyl alcohol) using a single staircase, in which the experimenter presented 3 pens to the participant (1 with a dilution of the odorant and 2 with the solvent). Using a forced choice design, we then instructed the participant to identify the odor-containing pen. We increased concentrations of the odorant when the odor was not correctly identified and decreased when the odor was correctly identified twice in a row. We defined the threshold as the mean of the 4 last reversals points (out of 7) of the staircase, leading to a score range from 1 to 16. For the discrimination task, we presented 3 odor-containing pens to the participant, with 2 pens containing the same odorant and a third pen containing a different odorant. Using a forced-choice design, we then asked participants to identify which of the three smelled different. We added up the number of correct identifications, leading to a score range from 0 to 16. For odor identification, the experimenter presented 16 common odorants to the participant and asked him/her to choose from a list of 4 descriptors. Again, we added up the number of correct identifications leading to a score range from 0 to 16. As a standard procedure, we obtained a global olfactory score by calculating the sum of scores of the three subtests (i.e., Threshold, Discrimination and Identification, TDI score; range: 1–48), for which normative values are available to classify participants in terms of functional anosmia (TDI < 16), hyposmia (between 16 and 30.3) and normosmia (Hummel et al., 2007).

### 2.3 MRI data acquisition

We acquired MRI data on a 3.0 Tesla Prisma Fit MRI scanner (Siemens Magnetom) using a 32-channel head coil, at the Functional Neuroimaging Unit of the research center at the Institut Universitaire de Gériatrie de Montréal (IUGM). Specifically, we acquired T2-weighted images in Turbo Spin Echo mode and 29 coronal slices of 2 mm were acquired with the following parameters: voxel size: 0.2 * 0.2 * 2.0 mm, repetition time: 6100.0 ms, echo time: 83 ms, field of view: 140 mm, flip angle: 150 deg., as previously described for olfactory bulb volumetry (Huart et al., 2013, Seubert et al., 2013). Total scanning session lasted one hour and included both structural and functional scans, results on functional connectivity within the chemosensory system are published elsewhere (Tremblay et al., 2020).

### 2.4 Olfactory bulb volume

**Fig. 1:**
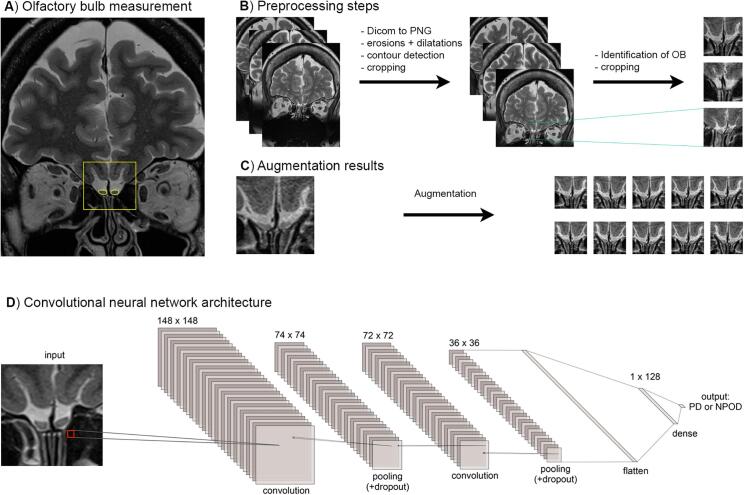
A) Example of the olfactory bulb manual measurement and region selected for the CNN (as measured in yellow) on T2-weighted scans. B) Preprocessing steps applied on the original scans C) Example of data augmentation. D) Architecture of the convolutional neural network. OB = olfactory bulb, PD = Parkinson’s disease, NPOD = Non-parkinsonian olfactory dysfunction. (For interpretation of the references to color in this figure legend, the reader is referred to the web version of this article.)

We measured olfactory bulb volumes as previously described (Hummel et al., 2015, Rombaux et al., 2009a, Yousem et al., 1997) using MIPAV 9.0 (Medical Image Processing, Analysis, and Visualization) software package (Center for Information Technology, National Institutes of Health). We carried out planimetric contouring and drew boundaries of the left and right olfactory bulbs manually on each coronal slice. We considered the first anterior slice in which the olfactory bulb becomes visible as the first slice, and the sudden decrease in olfactory bulb diameter that marks the beginning of the olfactory tract as the last slice. We then added all drawn surfaces of each slice and multiplied them by the slice thickness (2 mm) to obtain a volume in mm^3^ (Fig. 1A).

Information on the subjects’ group and olfactory score was concealed from the experimenter who measured the olfactory bulb volume. All volumes were measured at least twice by the same experimenter. When the difference between the two measurements was less than 10% of their average, we calculated the mean of both measurements which we then used in statistical analysis. If the difference was more than 10%, we carried out a third measurement, which was the case for 10 participants including 4 controls, 4 patients with NPOD and 2 PD patients, and used the two closest measures.

### 2.5 Statistical analysis

We performed statistical analysis of behavioral data and olfactory bulb volume using SPSS software (IBM SPSS Statistics 23.0). To compare the olfactory bulb volume between groups, we computed repeated measures ANOVA with *group* (3 levels: PD, NPOD, controls) as between subject factor and *side* (2 levels: right olfactory bulb volume, left olfactory bulb volume) as within subject variable. Then, we calculated univariate ANOVA analyses for each variable. Finally, we calculated Pearson correlation coefficients between volumetric measures of the olfactory bulb, olfactory function, and PD patients’ clinical data. For all multiple comparisons we applied Bonferroni corrections. We set the level of significance at *p* < 0.05.

### 2.6 Machine learning

#### 2.6.1 Image pre-processing and augmentation

All T2-weighted MRI scans from PD and NPOD patients (n = 197; 105 PD, 92 NPOD) that were used for the olfactory bulb measurements (6.6 ± 1.1 scans per patient; range: 5–9) were converted from DICOM format to PNG format using the Pydicom package (https://pypi.org/project/pydicom/; Mason, 2011), prior to further processing. We cropped scans of an original size of 672 × 896 pixels to a size of 150 × 150 pixels, so that the scans included the olfactory bulb and its surrounding area (Fig. 1A). Given the variance in the relative position of the olfactory bulb across subjects, we grouped scans based on the position of the olfactory bulb, and automatically processed and cropped these scans. See Fig. 1B for an example of the pre-processing steps and anatomically relevant regions selected.

To increase the number of scans, we applied image augmentation techniques including random rotation (angle = 5 deg.), width shifting (upper bound = 5%), height shifting (upper bound = 5%), rescaling, shear transformation (shear intensity = 0.05) and horizontal flipping to all scans (Nalepa et al., 2019) (Fig. 1C). Upon augmentation, we generated a total of 25 images from each original scan for a final number of 4925 scans (PD: 2625, 53.3%; NPOD: 2300, 46.7%). Augmented scans were split at the subject-level into training, validation and test sets so that all scans of one subject only appear in one set. Scans of 18 (9 PD, 9 NPOD), 6 (3 PD, 3 NPOD) and 6 subjects (3 PD, 3 NPOD) were randomly assigned to the training, validation and test sets, respectively.

#### 2.6.2 Convolutional neural network

We used a CNN to classify scans as either being from a PD or a NPOD patient. The CNN comprised 7 layers including 2 convolutional layers, 2 subsampling layers, 1 flatten layer and 2 fully connected layers. The number of trainable parameters of the CNN was 5,318,946. Both convolutional layers had 32 convolution kernels and used a kernel size of 3 × 3 pixels. We set stride to 1 along both height and width, and padding was not used. The subsampling layers performed 2 × 2 max pooling operations with a stride of 2 to reduce the dimension of the feature maps. We applied dropout after each subsampling layer with a probability of 50% (Srivastava et al., 2014). We used a fully connected layer of 128 units, followed by dropout regularization of 50% probability and then a final output layer (Fig. 1D).

**Supplementary Fig. 1:**
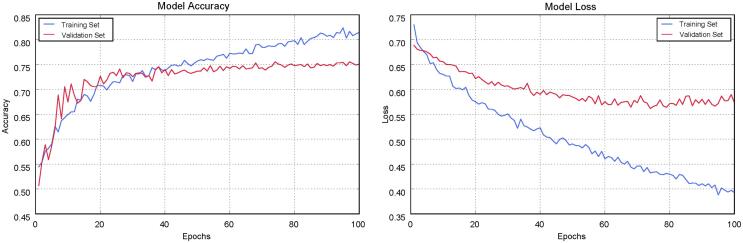
Performance of the convolutional neural network, as indicated by accuracy and loss per epoch, over the course of model training.

We used the Python programming language (version 3.7.5) and the Keras library (version 2.2.4) for the implementation and validation of the CNN. The CNN was trained with a batch size of 32 for a total of 100 epochs, using a stochastic gradient descent optimizer (learning rate = 0.0003, momentum = 0.0). We monitored the accuracy and loss incurred during the training of CNN over time across epochs (Supplementary Fig. 1). For visualizing and highlighting the relevant regions associated with the model’s output, we used Gradient-weighted Class Activation Mapping (Grad-CAM), a generalized form of Class Activation Mapping (CAM) based on the calculation of gradients from neurons of the last convolution layer of the neural network (Selvaraju et al., 2017). We superimposed all correctly identified scans and their corresponding Grad-CAM activation map, and we normalized them so that the position of the olfactory bulb would be at the same position for all these scans. We then extracted the class discriminative regions and presented them on a white background to highlight the regions associated with the CNN’s output (Fig. 5).

#### 2.6.3 Performance metrics

We used multiple performance metrices including accuracy, precision (i.e., positive predictive value, PPV), recall (i.e., sensitivity or true positive rate, TPR), specificity (i.e., true negative rate, TNR) and F1 score to systematically assess the performance of the CNN in differentiating PD and NPOD (Table 2). We further computed confusion matrices depicting true positive (TP), true negative (TN), false negative (FN) and false positive (FP) in the classification of PD and NPOD patients, for training and test sets.

**Table 2:** Scan-level performance of the CNN when classifying the training, and test samples.

	Accuracy	Loss	Precision (PPV)	Recall (Sensitivity)	Specificity	F1 score
Training	86.3%	0.4127	89.4%	85.4%	87.5%	87.4%
Test	88.3%	0.3365	88.2%	88.4%	88.2%	88.3%

PPV: positive predictive value.

Accuracy is defined as the ratio of total number of predictions that are correct, and calculated using the proportion of the true positive and true negative values:

$$\mathrm{accuracy}=\frac{\mathrm{TP}+TN}{\mathrm{TP}+TN+FP+FN}$$

Precision is the proportion of true positive values among all the values predicted to be positive, it represents how many of the selected items are relevant:

$$\mathrm{precision}=PPV=\frac{\mathrm{TP}}{\mathrm{TP}+FP}$$

Recall is the proportion of trues positive values that are correctly identified as such, it represents how many of the relevant items are selected:

$$\mathrm{recall}=sensitivity=TPR=\frac{\mathrm{TP}}{\mathrm{TP}+FN}$$

Specificity measures the proportion of the true negatives that are correctly identified as such:

$$\mathrm{specificity}=TNR=\frac{\mathrm{TN}}{\mathrm{TN}+FP}$$

The F1 score is the harmonic mean of the precision and recall:

$$F1\;score=2\times \;\frac{\mathrm{precision}\times recall}{\mathrm{precision}+recall}$$

## 3 Results

### 3.1 Behavioral results

Olfactory performance, as measured by the TDI score, revealed a significant effect of *group* [F(2,42) = 44.51; p < .001, η_p_^2^ = 0.68]. Same results were obtained for each sub-test separately as an effect of *group* was found for threshold [F(2,42) = 51.30; p < .001, η_p_^2^ = 0.71], discrimination [F(2,42) = 16.76; p < .001, η_p_^2^ = 0.44] and identification [F(2,42) = 29.37; p < .001, η_p_^2^ = 0.58] *Post hoc* comparisons confirmed significantly decreased olfactory function (TDI, Threshold, Discrimination and Identification) in both PD patients and patients with NPOD when compared to control participants (both p < .001), while no significant differences between PD and NPOD patients were observed.

Next, no group differences were found for cognitive function (MoCA) [F(2, 42) = 0.04; p = .96]. However, there were a significant group difference with respect to depressive symptoms (BDI) [F(2, 42) = 11.32; p < .001, η_p_^2^ = 0.35], with PD patients being significantly more depressed than both controls (p < .001) and NPOD patients (p = .003)

### 3.2 Olfactory bulb volume

**Fig. 2:**
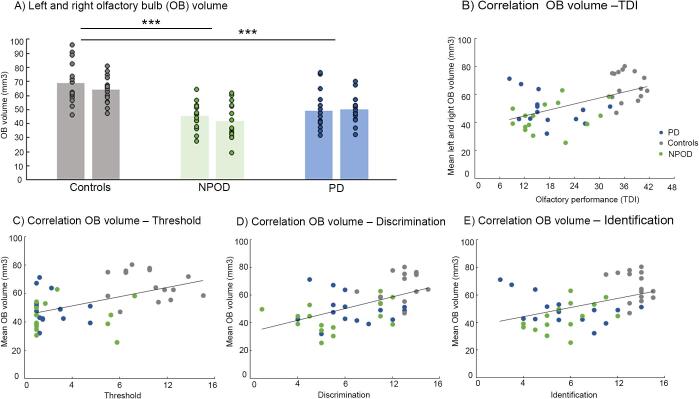
A) Inter-group comparison of average volumes of the left and right olfactory bulb respectively. Each dot represents one subject. B) Correlation between the average left–right olfactory bulb volume and olfactory performance as measured by the TDI score (Threshold, Discrimination, and Identification tests) of the 3 groups, and separately for C) threshold, D) discrimination and E) identification score. OB = olfactory bulb, PD = Parkinson’s disease, NPOD = Non-parkinsonian olfactory dysfunction.

For the olfactory bulb volume analysis, ANOVA revealed a main effect of *group* [F(2,42) = 18.49; p < .001, η_p_^2^ = 0.47], but we did not find an effect of left/right *side* [F(1,42) = 2.39; p = .13] nor an interaction between *side* and *group* [F(2,42) = 1.06; p = .36]. Separate univariate ANOVA confirmed an effect of *group* for both the right [F(2,42) = 14.09; p < .001, η_p_^2^ = 0.40] and the left olfactory bulb [F(2,42) = 15.98; p < .001, η_p_^2^ = 0.43]. *Post hoc* test confirmed a significantly smaller bulb volume in both PD patients (right: p < .001; left: p < .001) and NPOD patients (right: p = .003; left: p < .001) when compared to controls. Again, we did not find any differences between PD patients and NPOD patients (Fig. 2A).

Further, when analyzing all participants together we found a significant correlation between olfactory bulb volume and global olfactory score (right: r = 0.492, p = .015; left: r = 0.517, p < .001, mean right-left volume: r = 0.538, p < .001), as well as for all the subtests, namely threshold (right: r = 0.473, p = .015; left: r = 0.474, p = .015, mean right-left volume: r = .506p < .001), discrimination (right: r = 0.466, p = .015; left: r = 0.531, p < .001, mean right-left volume: r = 0.530, p < .001), but for identification we found only a correlation for the left side and the mean olfactory bulb volume (right: r = 0.387, p = .135; left: r = 0.444, p = .03, mean right-left volume: r = 0.442, p = .03) (Fig. 2).

When we analyzed the PD patients’ group on its own, we found no correlation between olfactory bulb volume (right, left or mean right-left volume) and disease duration, medication (LEDD), cognitive MoCA score, BDI score, disease stage (H&Y), or asymmetry of symptoms (right/left).

### 3.3 Results of CNN in the classification of PD and NPOD patients

**Fig. 3:**
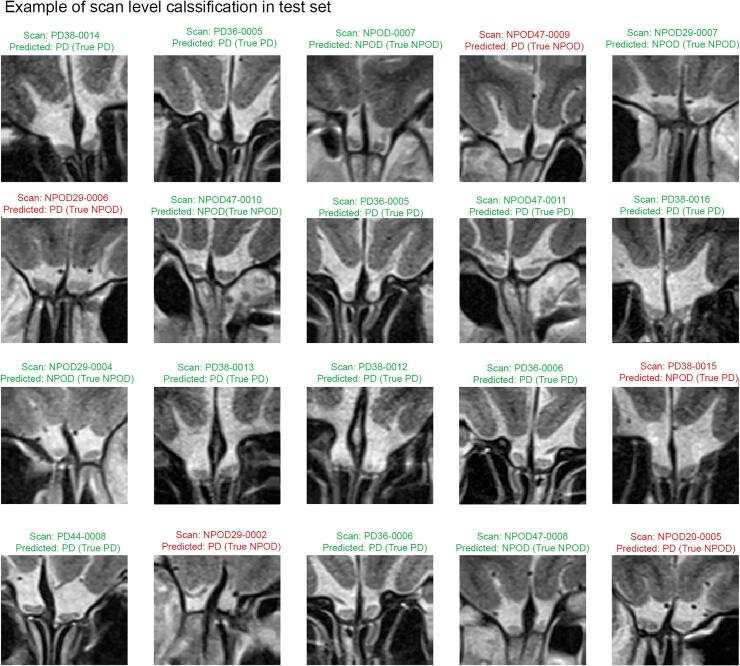
Example of scan-level classification of the test set. The 20 scans shown were not seen by the CNN previously. Green: correctly predicted, red: incorrectly predicted. PD = Parkinson’s disease, NPOD = Non-parkinsonian olfactory dysfunction. (For interpretation of the references to color in this figure legend, the reader is referred to the web version of this article.)

**Fig. 4:**
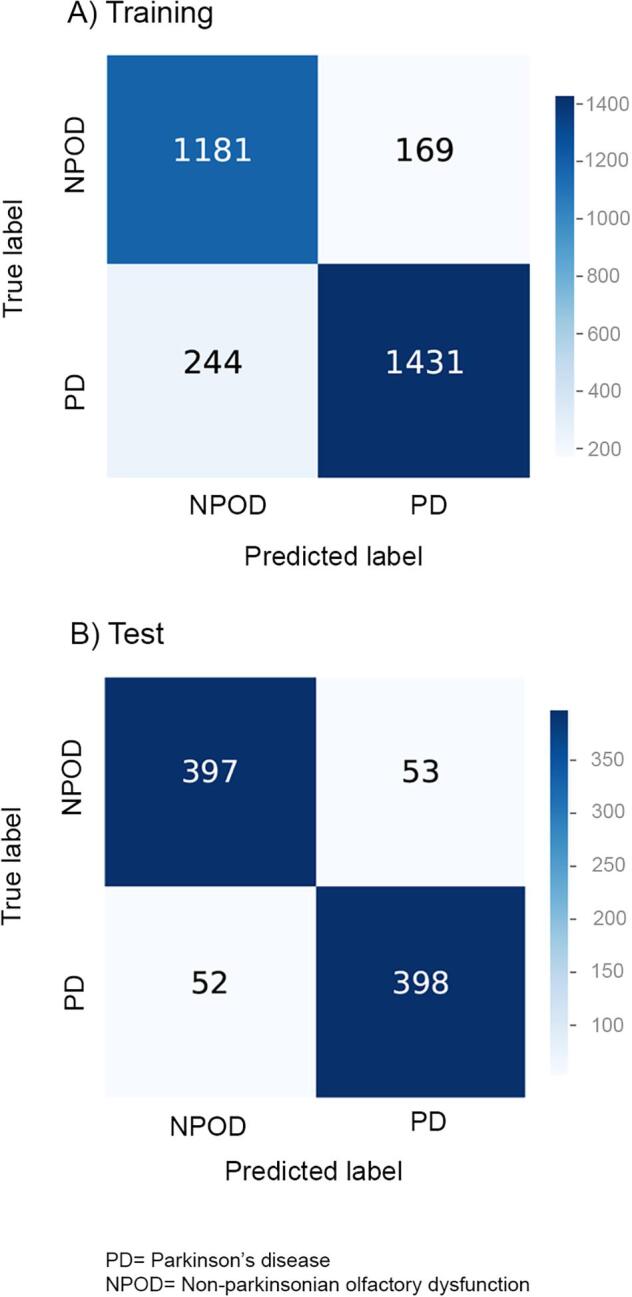
Confusion matrices of the CNN representing the number of correctly and incorrectly predicted scans in the classification of patients with Parkinson’s disease (PD) and patients with non-parkinsonian olfactory dysfunction (NPOD) in the training and test sets.

We observed a training accuracy of 86.3% and a test accuracy of 88.3% when classifying PD patients from NPOD patients. Precision, recall, specificity and F1 score are presented in Table 2. An example of scan-level classification is shown in Fig. 3. Confusion matrices illustrating the predicted label and true label of the CNN for the binary classification task are presented in Fig. 4. According to the confusion matrices, the CNN model led to (a) a correct diagnosis of 2612 scans (86.3%) and a misdiagnosis of 413 scans (244 PD and 169 NPOD) out of 3025 scans of the training set, and (b) a correct diagnosis of 795 (88.3%) scans and a misdiagnosis of 105 scans (52 PD, 53 NPOD) out of 900 scans of the test set (Fig. 4)

**Fig. 5:**
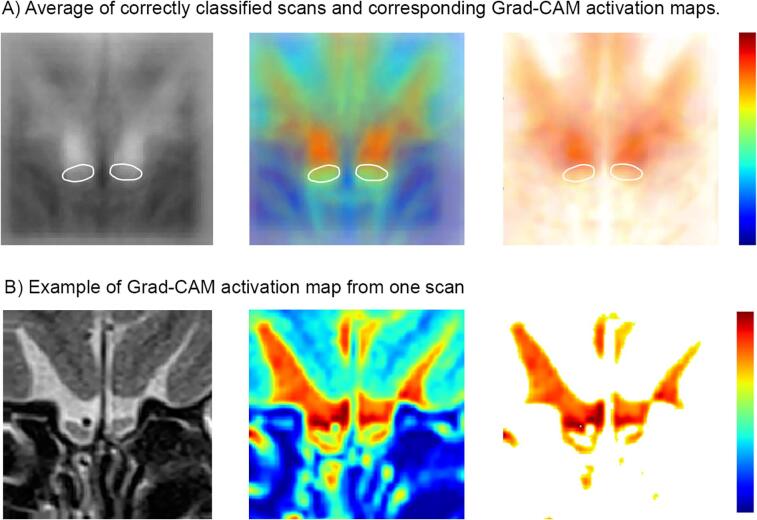
A) Average of correctly classified scans of the test set (left) with superimposition of the Grad-CAM activation maps (middle) and the class-discriminative regions on a white background (right). Grad-CAM activation map shows the class-discriminative regions and the red color corresponds to higher relevance to the CNN’s output. The approximate position of the olfactory bulb is indicated by the white ovals. B) Individual example of one scan and the corresponding Grad-CAM activation map. (For interpretation of the references to color in this figure legend, the reader is referred to the web version of this article.)

The average of the correctly identified scans and the corresponding Grad-CAM depicting the class-discriminative regions (Fig. 5) shows that the olfactory bulb and its surrounding areas, especially the region above the olfactory bulb that extend into the olfactory sulcus are more looked at by the CNN to discriminate between groups.

### 3.4 Data availability statement

Data will be made available on the database of the University of Quebec at Trois-Rivières upon publication.

## 4 Discussion

In the present study, we report the results of an investigation of olfactory bulb structure and surrounding areas in PD patients when specifically compared to patients with non-parkinsonian olfactory dysfunctions (NPOD) and healthy control participants. Our results confirm the presence of a reduced olfactory bulb volume in both PD patients and NPOD patients when compared to control participants. While manual measures from olfactory bulb were not able to differentiate PD patients from NPOD patients, a CNN applied to scans of the olfactory bulb and its surroundings yielded a training accuracy of 86.3% and a test accuracy of 88.3% in the discrimination between PD and NPOD patients.

The result of a reduced olfactory bulb volume in PD patients is concordant with previous studies (Brodoehl et al., 2012, Chen et al., 2014, Hang et al., 2015, Li et al., 2016, Tanik et al., 2016, Wang et al., 2011) and in line with various alterations of the olfactory bulb including neuronal loss (Pearce et al., 1995). Even though there are some inconstancies as several studies did not show significant decrease in olfactory bulb volume in PD patients when compared to control participants (Altinayar et al., 2014, Hakyemez et al., 2013, Hummel et al., 2010, Mueller et al., 2005a, Paschen et al., 2015), our results confirmed that the olfactory bulb volume is indeed reduced in PD patients, in line with a meta-analysis (Li et al., 2016). Similarly, NPOD patients, including post-viral OD and sinonasal OD, exhibited reduced olfactory bulb volume when compared to controls, which is also consistent with published literature (Gudziol et al., 2009, Han et al., 2017, Mueller et al., 2005b, Rombaux et al., 2006a, Rombaux et al., 2008). Nevertheless, we did not identify any differences between olfactory bulb volumes of PD and NPOD patients, when using manual volumetric measurements. In fact, although the underlying pathomechanisms of reduced olfactory bulb volume in PD and NPOD are different, both groups showed a similar degree of olfactory loss, as found in our participants and reported by other studies (Tremblay et al., 2017, Whitcroft et al., 2017). Further, we found no correlation between olfactory bulb volume and disease stage or severity which is in line with previous reports that did not show any correlation with either disease stage, disease duration, age of disease onset, lateralization of initial motor symptoms, left–right limb motion function score or cognitive status in PD (Altinayar et al., 2014, Brodoehl et al., 2012, Chen et al., 2014, Mueller et al., 2005a, Paschen et al., 2015). Our results are in line with the notion of olfactory bulb volume to be a neuroanatomical correlate of olfactory function (Buschhuter et al., 2008, Hummel et al., 2015), independently of any underlying condition that may affect its volume. Interestingly, a recent study however suggests that overall chemosensory function such as flavor perception may be one method to behaviorally distinguish between PD and NPOD patients (Aubry-Lafontaine et al., 2020).

In contrast to manual measurements, when applying deep learning models to the same scans, our model was able to correctly discriminate between PD patients and NPOD patients with an accuracy of 88.3%, a recall of 88.4% and a precision of 88.2%, when MR images of the test set were classified by the CNN. Taken together, these results may comprise a crucial step towards the development of early diagnostic tools of PD based on OD and they emphasize the potential of such algorithms to extract information from medical images of patients with OD. Future studies should investigate if similar techniques can be used in outcome prediction of subjects with a high risk to develop PD, such as patients suffering from rapid eye movement (REM) sleep behavioral disorder (RBD), characterized by loss of normal atonia of REM sleep, in which up to 90% of patients will develop PD (Postuma et al., 2015). Other non-motor symptoms of PD should also be taken into consideration because of their association with OD and their potential in predicting the development of PD (Masala et al., 2018).

With the aim of trying to better understand which features of the scans the CNN were using to differentiate between groups, we averaged the Grad-CAM activation maps of the correctly classified scans of the test set (Fig. 5). Even though these images are blurry as a result of averaging across multiple scans, and therefore must be interpreted carefully, it still gives us information regarding the features that are the most looked at by the CNN in the classification. These regions include the olfactory bulb and its boundary, and more importantly, the area right above the olfactory bulb that extends into the olfactory sulcus. Therefore, we hypothesize the CNN to also rely on additional adjacent orbitofrontal cerebral structures such as (a) the olfactory sulcus, (b) the gyrus rectus, and (c) and the medial orbitofrontal cortex (OFC). All these structures are known to be involved in olfactory processing (Lundstrom et al., 2011) and the size of these structures reflects olfactory function, in analogy to the olfactory bulb. In fact, a correlation between olfactory function and the depth of the right olfactory sulcus (Hummel et al., 2003), the thickness and volume the medial OFC and the area around the olfactory sulcus (Frasnelli et al., 2010) as well as the of the gyrus rectus (Delon-Martin et al., 2013) were reported. Further, two studies reported significantly swallower olfactory sulci in PD patients when compared to healthy controls (Tanik et al., 2016, Wang et al., 2011), even though another study reported no difference (Hang et al., 2015), and no correlations between the olfactory sulcus depth and olfactory function (Hang et al., 2015, Wang et al., 2011). Nevertheless, these are comparisons between PD patients and healthy control participants, and thus not with participants showing similar olfactory function as PD patients. Reduced olfactory sulcus along with frontal cortical atrophy as reported in PD (Lee et al., 2014) might lead to a greater space between the olfactory bulb and the frontal cortex. However, cortical atrophy was also reported in patients with anosmia and hyposmia from various etiology when compared to controls (Bitter et al., 2010a, Bitter et al., 2010b). To date, no direct comparison in cortical volume or density was made between PD patients and patients with NPOD. Olfactory bulb related measurement is of interest in PD patients and may give more information on the underlying pathology as the olfactory bulb volume was found to be correlated with the putamen volume and the olfactory sulcus correlated to the hippocampal volume (Tanik et al., 2016). Further investigating the olfactory bulb and sulcus and most importantly the space between the olfactory bulb and the above cortex, by developing an approach to measure this specific region, might bring further insight into the study of PD-related olfactory loss and may help to differentiate PD-related OD from other forms of OD in early stages of the disease.

We acknowledge that we tested a limited number of participants and these results must be interpreted with care. The total number of scans used in the present study is small for deep learning techniques and the model may not generalize well to additional clinical data. Thus, the model should be trained and validated with more data to be potentially used as an assistive diagnostic tool in the future. Further, all scans used in the manual measurement of the olfactory bulb volume were used to train or test the CNN to maximize the number of scans, therefore some scans in which the visualization of olfactory bulb was not optimal (for instance, scans depicting more distal or proximal parts of the olfactory bulb) may have confounded the results, leading to compromised accuracy. In addition, we did not have access to the participants’ UPDRS scores which would have been interesting to better characterize the included PD population and to further assess potential correlation with disease severity, even though we found no correlation with other clinical data. Future studies should be conducted to validate these results in larger sample sizes and in better characterized cohorts including patients with prodromal PD. To do so, T2-weighted scans of the olfactory bulb should be included in existing research and clinical protocols.

In conclusion, olfactory bulb volume was found to be reduced in both PD-related OD and NPOD when compared to control participants, but was not different between PD and NPOD patients. In the meantime, considerable accuracy was achieved when a CNN was used to differentiate olfactory bulb scans from PD and NPOD patients, and therefore may lead to refined early diagnosis, although this approach still requires further validation.

## Declaration of Competing Interest

The authors declare that they have no known competing financial interests or personal relationships that could have appeared to influence the work reported in this paper.
